# Frequency of Modification of Pharmacological Treatment Is Equivalent for Virtual and In-Person Psychiatric Visits

**DOI:** 10.1089/tmr.2023.0004

**Published:** 2023-03-22

**Authors:** Amal Mumtaz, Aisha O. Adigun, Rif S. El-Mallakh

**Affiliations:** Mood Disorders Research Program, Department of Psychiatry and Behavioral Sciences, Depression Center, University of Louisville School of Medicine, Louisville, Kentucky.

**Keywords:** medication modification, outcomes, psychiatry, quality, telepsychiatry, virtual visits

## Abstract

**Background:**

During the coronavirus pandemic there was a rapid adoption of telehealth services in psychiatry, which now accounts for 40% of all visits. There is a dearth of information about the relative efficacy of virtual and in-person psychiatric evaluations.

**Methods:**

We examined the rate of medication changes during virtual and in-person visits as a proxy for the equivalence of clinical decision-making.

**Results:**

A total of 280 visits among 173 patients were evaluated. The majority of these visits were telehealth (224, 80%). There were 96 medication changes among the telehealth visits (42.8%) and 21 among the in-person visits (37.5%) (*z* = −1.4, *p* = 0.16).

**Conclusion:**

Clinicians were equally as likely to order a medication change if they saw their patient virtually or in person. This suggests that remote assessments yielded similar conclusions to in-person assessments.

## Introduction

In December 2019, visitors to Huanan and Yangchahu markets in Wuhan and racoon dogs being sold there developed an illness that was subsequently determined to be caused by the severe acute respiratory syndrome–related coronavirus.^1^ In the spring of 2020, the World Health Organization declared that the coronavirus 2019 (CoViD-19) outbreak was a pandemic.^2^ As the rate of CoViD-19 infections, severe illnesses, and deaths increased, many countries, including the United States, began declaring shutdowns and lockdowns.^3^ These shutdowns included nonemergency medical services. At that time only a fraction of states allowed parity of telehealth and in-person visits,^3^ and its use was predominantly limited to rural settings and about 8% of medical provider visits.^4^ By the spring of 2021, about one quarter of Americans were receiving telehealth services,^5^ a 200% increase.

Even before the CoViD-19 lockdown, there had been a call to digitalize psychiatric outpatient care.^6^ Nonetheless, psychiatrists had been concerned that use of virtual settings would lead to deterioration of the doctor–patient relationship, which many believe is central to the practice of psychiatry.^7–9^ Pre-CoViD-19 studies comparing in-person care with telepsychiatry generally showed that remote treatment was comparable or superior to in-person treatment.^10^ Patient satisfaction was generally good,^10^ but some patients felt a lesser connection to their clinician.^11^ Provider satisfaction is more mixed with concerns about quality of services, patient perceptions, and technological issues.^10^ Throughout CoViD-19 restrictions, clinician satisfaction with telepsychiatry effected adoption rates when an option was available.^12,13^

However, participants in pre-CoViD-19 studies chose to be involved in a telehealth study. Patients and clinicians through the CoViD-19 shutdowns had telehealth “forced” upon them. That difference is a potentially important variable.

We performed a quality assurance study examining the objective outcome of medication intervention comparing individuals who attended the same clinic remotely or in person. The study was performed after the shutdown restrictions were slowly being lifted in 2021, and some patients were transitioning back to in-person visits.

## Methods

The study was a quality assessment of visits at the University of Louisville outpatient psychiatric service. With the switch to virtual visits we needed to examine a quality measure to ensure that we were providing adequate care to our patients. We examined the rate of medication changes during virtual and in-person visits as a proxy for the equivalence of clinical decision-making because this method was utilized previously.^14^ All patients were ≥18 years. We identified times when patients came to two consecutive visits with the same provider and the same primary diagnosis and evaluated treatment changes on the second visit. We documented when there was a change in prescribed medication or dosage but excluded “as needed” (PRN or *Pro Re Nata*) medications. A test evaluating differences in proportions was used.^15^ Because this was a quality assessment study, it did not require evaluation or approval by the Human Subjects Protections Program of the University of Louisville.

## Results

The study period spanned all of 2021. A total of 280 visits among 173 patients were evaluated (Table 1). There were some 20 providers in the clinic, all of whom did both in-person and virtual visits; we corrected for this by only examining patients who came to the same provider at least twice in a row. The majority of these visits were telehealth (224, 80%) (Table 1). There were 115 women (66.5%), 57 men (33%), and 1 unspecified gender. The age range was 19–89 years (Median age 43 years; mean age 45.4 years; interquartile range 24 years); the majority of patients (98 or 56.6%) were under the age of 50 years.

**Table 1.** Demographics of Patients Studied

	Total		Telemedicine		In person	
	Number of patients (%)	Number of visits^a^	Number of patients (%)	Number of visits^a^	Number of patients (%)	Number of visits^a^
Total	173 (100)	280	142	224	37	56
Gender						
Male	57 (33.0)	88	45	65	13	23
Female	115 (66.0)	191	96	158	24	33
Unspecified	1 (0.005)	1	1	1	0	0
Age group						
18–29	28 (16.2)	52	26 (18.3)	48	3 (8.1)	4
30–39	39 (22.5)	63	36 (25.4)	56	4 (10.8)	7
40–49	31 (17.9)	49	25 (17.6)	39	7 (18.9)	10
50–59	38 (22.0)	63	29 (20.4)	47	10 (27.0)	16
≥60	37 (21.4)	53	26 (18.3)	34	13 (35.1)	19

^a^ Note that all “visits” are actually two consecutive visits with changes recorded on the second visit.

The primary diagnoses were major depressive disorder (*n* = 69 or 39.9%), generalized anxiety disorder (*n* = 66 or 38.2%), post-traumatic stress disorder (*n* = 44 or 25.4%), and attention-deficit/hyperactivity disorder (*n* = 33 or 19.1%). Of the encounters, 224 (80%) were virtual, and 56 (20%) were in person (Table 1). There were 118 visits with medication changes. Most medication changes involved dose change only (57%), and the remainder involved adding or discontinuing medications (43%). There were 96 medication changes among the telehealth visits (42.8%) and 21 among the in-person visits (37.5%). The difference was not significant (*z* = −1.4, *p* = 0.16).

## Discussion

We performed this study because there is a dearth of information regarding the relative efficacy of telehealth visits when patients do not choose that modality. We examined medication change as a proxy for clinical decision-making by psychiatrists in an academic outpatient clinic. Previous study had suggested that this was a reasonable measure of clinical decision-making.^14^ In our quality assurance examination, clinicians, were equally likely to act on their collected clinical data by ordering a medication change if they saw their patient virtually or in person. This suggests that remote assessments yielded similar conclusions to in-person assessments, even in patients who do not voluntarily participate in a telehealth study.

It is important to note that this study does not address the quality of care provided or health care outcomes. We did not measure patient satisfaction and did not collect any correlates of clinical outcomes. We utilized medication change as a measure because it is driven by patient complaints or clinician observations during patients' presentations.^16^ Thus, it reflected the quality of the transfer of information during the session. The lack of significant difference suggests that the quality of information transfer is equivalent, or nearly so, in the two forms of evaluation.

Telepsychiatry has grown considerably. During the height of the CoViD-19 restrictions, 40% of all outpatient visits were provided virtually by mental health providers, compared with only 11% by other providers.^17^ Even as CoViD-19 restrictions have been largely removed, and telepsychiatry continues to be highly utilized, accounting for 36% of all outpatient visits and 39% of all telehealth services.^17^

There are clear limitations to our study design. As noted earlier, we did not examine the actual quality of outcome. Furthermore, this was not a randomized study. Nearly all patients seen virtually did not have a choice of how the evaluation would be done, but all patients seen in person chose that format. Furthermore, it is possible that patients who would not do well with virtual visits simply dropped out of treatment during CoViD-19 restrictions and were not studied in our population.

Telepsychiatry will continue to be a growing presence in future health care. Additional studies need to be done to confirm the equivalence of remote versus in-person outcomes. Nonetheless, the early results of our study and the current literature suggest that using telehealth for mental health conditions is a reasonable option.

## Abbreviations Used

CoViD-19: coronavirus 2019
PRN: *Pro Re Nata*

## References

[1] Worobey M. Dissecting the early COVID-19 cases in Wuhan: Elucidating the origin of the pandemic requires understanding of the Wuhan outbreak. Science 2021;374(6572):1202–1204; doi: 10.1126/SCIENCE.ABM445 34793199

[2] Cucinotta D, Vanelli M. WHO declares COVID-19 a pandemic. Acta Biomed 2020;91(1):157–160. 32191675 10.23750/abm.v91i1.9397 PMC7569573

[3] Bergquist S, Otten T, Sarich N. COVID-19 pandemic in the United States. Health Policy Technol 2020;9(4):623–638. 32874854 10.1016/j.hlpt.2020.08.007 PMC7451131

[4] Mann DM, Chen J, Chunara R, et al. COVID-19 transforms health care through telemedicine: Evidence from the field. J Am Med Informatics Assoc 2020;27(7):1132–1135. 10.1093/jamia/ocaa072 PMC7188161 32324855

[5] Karimi M, Lee EC, Couture SJ, et al. National trends in telehealth use in 2021: Disparities in utilization and audio vs. video services. (Research Report No. HP-2022-04). Office of the Assistant Secretary for Planning and Evaluation, U. S. Department of Health and Human Services. February 2022. Available from: https://aspe.hhs.gov/sites/default/files/documents/4e1853c0b4885112b2994680a58af9ed/telehealth-hps-ib.pdf [Last accessed: August 7, 2022].

[6] Bender E. New APA resource addresses psychiatry in underserved areas. Psychiat News. 7 March 2008. Available from: https://psychnews.psychiatryonline.org/doi/full/10.1176/pn.43.5.0017 [Last accessed: June 20, 2022].

[7] Tasman A. Presidential address: The doctor-patient relationship. Am J Psychiatry 2000;157:1762–1768. 11058471 10.1176/appi.ajp.157.11.1762

[8] May C, Gask L, Atkinson T, et al. Resisting and promoting new technologies in clinical practice: The case of telepsychiatry. Soc Sci Med 2001;52:1889–1901. 11352414 10.1016/s0277-9536(00)00305-1

[9] Wagnild G, Leenknecht C, Sauher J. Psychiatrists' satisfaction with telepsychiatry. Telemed e-Health 2006;12:546–551. 10.1089/tmj.2006.12.546 17042708

[10] Hubley S, Lynch SB, Schneck C, et al. Review of key telepsychiatry outcomes. World J Psychiatry 2016;6(2):269–282. 27354970 10.5498/wjp.v6.i2.269 PMC4919267

[11] Urness D, Wass M, Gordon A, et al. Client acceptability and quality of life—Telepsychiatry compared to in-person consultation. J Telemed Telecare 2006;12:251–254. 16848938 10.1258/135763306777889028

[12] Parikh SV, Taubman DS, Grambeau M, et al. Going virtual during a pandemic: An academic psychiatry department's experience with telepsychiatry. Psychopharmacol Bull 2021;51(1):59–68. 33897063 10.64719/pb.4392 PMC8063128

[13] Connolly SL, Miller CJ, Gifford AL, et al. Perceptions and use of telehealth among mental health, primary, and specialty care clinicians during the COVID-19 pandemic. JAMA Netw Open 2022;5(6):e2216401. 35671053 10.1001/jamanetworkopen.2022.16401 PMC9175071

[14] Alleyne JE, Bashir AS, Birdwhistell ML, et al. The effect of teaching clinics on prescribing practices. Acad Psychiatry 2015;40(2):317–320. 26122354 10.1007/s40596-015-0385-y

[15] El-Mallakh RS, Cowdry RW, Pettigrew IE. Evaluating change: A simple technique for determining statistical significance of proportional criteria. J Healthcare Qual 1994;16:14–17. 10.1111/j.1945-1474.1994.tb00681.x 10135767

[16] Hodgkin D, Stewart MT, Merrick EL, et al. Prevalence and predictors of physician recommendations for medication adjustment in bipolar disorder treatment. J Affect Disord 2018;238:666–673. 29966931 10.1016/j.jad.2018.06.012 PMC13181833

[17] Lo J, Rae M, Amin K, et al. Telehealth has played an outsized role meeting mental health needs during the COVID-19 pandemic. Kaiser Family Foundation, Published: Mar 15, 2022. Available from: https://www.kff.org/coronavirus-covid-19/issue-brief/telehealth-has-played-an-outsized-role-meeting-mental-health-needs-during-the-covid-19-pandemic/ [Last accessed: August 13, 2022].

